# Data on cellular lipids of *Yarrowia lipolytica* grown on fatty substrates

**DOI:** 10.1016/j.dib.2018.10.116

**Published:** 2018-10-28

**Authors:** Alexandra Daskalaki, Ioanna A. Vasiliadou, Stamatia Bellou, Ludwika Tomaszewska-Hetman, Chrisanthi Chatzikotoula, Barbara Kompoti, Seraphim Papanikolaou, Dimitris Vayenas, Stavros Pavlou, George Aggelis

**Affiliations:** a Unit of Microbiology, Division of Genetics, Cell and Developmental Biology, Department of Biology, University of Patras, 26504 Patras, Greece; b Laboratory of Food Microbiology & Biotechnology, Department of Food Science and Human Nutrition, Agricultural University of Athens, Athens, Greece; c Department of Chemical Engineering, University of Patras, 26504 Patras, Greece; d Foundation for Research and Technology Hellas, Institute of Chemical Engineering and High Temperature Chemical Processes, Stadiou str., Platani, 26504 Patras, Greece

**Keywords:** t (h), fermentation time, L, cellular lipid, x, cell mass, NL, neutral lipids, G+S, glycolipids plus sphingolipids, P, phospholipids

## Abstract

*Yarrowia lipolytica*, which is model oleaginous yeast with high industrial interest, was cultivated on fatty substrates. Data concerning fatty acid composition of both substrate and yeast lipids and comparisons of the experimental data with model predictions presented in “Biomodification of fats and oils and scenarios of adding value on renewable fatty materials through microbial fermentations: Modelling and trials with *Yarrowia lipolytica*” (Vasiliadou et al., 2018) were provided. Furthermore, the total yeast lipids were fractionated into their main fractions, that is, phospholipids, glucolipids plus sphingolipids and neutral lipids, and the fatty acid composition of each lipid fraction was reported.

**Specifications table**

**Table:** 

Subject area	*Biotechnology, Chemistry*
More specific subject area	*Lipid Biotechnology*
Type of data	*Tables, figures*
How data was acquired	*The yeast Yarrowia lipolytica was cultivated on fatty substrates and the fatty acid composition of both the extracellular and intracellular lipids, as well as of their fractions was determined using an Agilent 7890 A device Gas Chromatography (Agilent Technologies, Shanghai, China).*
Data format	*Raw samples were collected during growth of Y. lipolytica and processed. Substrate and cellular lipids were purified and analysed.*
Experimental factors	*Different fatty materials were used as substrates for Y. lipolytica.*
Experimental features	*Various fats of plant (i.e., olive, sunflower, palm and linseed) and animal (i.e., cod liver and beef tallow) origin were used as carbon substrates for Y. lipolytica. Cultures, carried out in 250-mL Erlenmeyer flasks, were incubated in a rotary shaker (ZHWY211C, Zhicheng, Shanghai, China) at 180 rpm and T=28±1 °C.*
Data source location	*University of Patras, Greece*
Data accessibility	*The data are available in this article*
Related research article	*[1]* Vasiliadou et al., 2018 *“Biomodification of fats and oils and scenarios of adding value on renewable fatty materials through microbial fermentations: Modelling and trials with Yarrowia lipolytica.” Journal of Cleaner Production, 200, 1111–1129.*

**Value of the data**

• The data can be used in order to identify the fatty acid specificity of *Yarrowia lipolytica.*
• The composition of lipids (i.e., mainly neutral) accumulated in *Y. lipolytica* can be pre-determined.
• New biomodification processes of common fats can be designed.

## 1 Data

The data article includes Table 1 reporting fatty acid composition of lipid fractions of *Yarrowia lipolytica* growing on olive oil, linseed oil, palm oil, sunflower oil, cod liver oil, and beef tallow, and two Figures showing: (1) Experimental data and theoretical predictions of the fatty acid composition of extracellular and intracellular lipids of *Y. lipolytica* and (2) theoretical fatty acid profiles of the free fatty acid fraction released in the growth medium during growth of *Y. lipolytica* on the above mentioned fatty substrates.

**Table 1:** Fatty acid composition of lipids accumulated in *Yarrowia lipolytica* growing on various fats of plant or animal origin.

Culture on olive oil									
t (h)	L/x %, w/w	Lipid fractions	% in total lipids	C16:0	C16:1	C18:0	C18:1	C18:2	Others
109	28.0	NL	94.0	7.3	5.6	1.2	70.0	15.1	0.7
		G + S	4.2	9.2	5.4	2.7	65.9	15.2	0.6
		P	1.9	11.7	7.7	0.7	44.0	35.7	0.4
335	13.0	NL	96.0	7.2	10.0	1.8	60.9	19.2	1.0
		G + S	2.7	14.6	10.9	1.2	52.5	18.9	1.9
		P	1.4	10.6	13.3	2.1	49.7	22.8	1.6

Culture on linseed oil										
t (h)	L/x %, w/w	Lipid fractions	% in total lipids	C16:0	C16:1	C18:0	C18:1	C18:2	C18:3α	Others
72	21.9	NL	86.5	4.3	1.6	1.3	15.1	18.9	58.6	0.2
		G + S	10.3	5.5	1.6	2.0	21.2	17.9	36.3	15.6
		P	3.3	16.1	2.8	2.4	25.5	20.9	24.7	7.6
263	12.2	NL	92.0	4.6	4.5	2.3	21.2	19.4	47.9	0.2
		G + S	4.6	10.2	5.4	7.5	24.8	16.8	35.0	0.4
		P	3.7	15.8	7.9	1.6	29.5	17.0	28.2	–

Culture on palm oil									
t (h)	L/x %, w/w	Lipid fractions	% in total lipids	C16:0	C16:1	C18:0	C18:1	C18:2	Others
67	28.5	NL	90.2	23.6	3.9	2.1	51.1	19.2	0.2
		G + S	5.5	23.8	1.9	3.8	39.1	15.8	15.6
		P	4.3	15.8	7.2	1.3	31.5	39.2	5.0
238	6.9	NL	94.7	21.2	6.0	5.5	44.3	22.7	0.4
		G + S	2.6	24.0	4.4	3.8	40.7	24.3	2.8
		P	2.8	15.8	7.2	1.3	31.5	39.2	5.0

Culture on sunflower oil									
t (h)	L/x %, w/w	Lipid fractions	% in total lipids	C16:0	C16:1	C18:0	C18:1	C18:2	Others
72	25.5	NL	87.5	5.2	1.9	2.8	30.9	55.7	3.4
		G + S	10.0	5.1	2.0	2.3	24.8	32.6	33.1
		P	2.5	13.2	6.1	1.7	28.0	41.6	9.3
357	4.6	NL	84.4	4.2	4.3	2.2	30.0	55.1	4.2
		G + S	11.8	9.2	3.4	4.5	24.4	25.1	33.3
		P	3.8	10.8	7.3	0.9	31.0	38.0	12.0

Culture on cod liver oil													
t (h)	L/x %,w/w	Lipid fractions	% in total lipids	C16:0	C16:1	C18:0	C18:1	C18:2	C18:3	C20:1	C20:5	C22:6	Others
72	25.5	NL	89.6	16.0	14.5	3.0	31.7	6.9	9.0	7.4	2.1	3.0	6.5
		G + S	7.8	7.6	5.5	2.2	18.3	7.6	4.2	16.6	–	3.9	34.1
		P	2.6	10.8	11.7	2.7	41.0	22.4	3.1	3.6	–	0.2	4.4
357	4.6	NL	86.0	11.1	17.2	3.4	37.7	10.1	2.5	5.4	0.5	0.7	11.5
		G+S	10.4	11.0	9.9	3.5	31.6	6.8	7.1	9.9	2.1	1.0	17.1
		P	3.6	8.1	13.8	1.5	44.7	21.7	3.0	–	–	0.5	6.7

Culture on beef tallow									
t (h)	L/x %,w/w	Lipid fractions	% in total lipids	C16:0	C16:1	C18:0	C18:1	C18:2	Others
96	2.1	NL	88.8	13.9	7.1	40.3	30.6	4.9	3.2
		G + S	5.6	15.7	4.5	32.3	22.8	4.9	19.8
		P	5.6	13.6	14.1	10.5	28.0	21.0	12.8
									
235	9.5	NL	95.5	15.3	9.9	26.4	36.6	7.3	4.5
		G + S	2.5	11.7	5.6	22.5	24.0	7.3	28.9
		P	2.0	12.9	12.7	3.4	37.3	24.8	9.0

Culture conditions: pH 6.0 ± 0.5; T = 28 °C; agitation rate 280 rpm. Data represent means of two replicates.

## 2 Experimental design, material, and methods

The yeast *Y. lipolytica* ACA-DC 50109 was used in the current investigation. The strain was maintained on potato dextrose agar (PDA, Conda, Madrid, Spain) at 7 ± 1 °C and re-cultured twice a month.

The growth media contained (in g/L): MgSO_4_.7H_2_O (Fluka, Steinheim, Germany), 1.5; KH_2_PO_4_ (Fluka), 7.0; Na_2_PO_4_ (Fluka), 2.0; CaCl_2_.2H_2_O (Carlo Erba, Rodano, Italy), 0.1; ΖnSO_4_.7H_2_O (Merck, Darmstadt, Germany), 0.001; CuSO_4_.5H_2_O (BDH, Poole, England), 0.0001; Co(NO_3_)_3_.3H_2_O (Merck), 0.0001; MnSO_4_.5H_2_O (Fluka), 0.0001; (NH_4_)_2_SO_4_ (Fluka), 0.5; yeast extract (Sigma, Steinheim, Germany), 2.0. Various commercial fats of plant (i.e., olive, sunflower, palm, and linseed) and animal (i.e., cod liver and beef tallow) origin were used as carbon and energy sources at a concentration of 25 g/L.

Experiments were performed in 250-mL Erlenmeyer flasks. The flasks containing 50±1 mL of growth media were sterilized at 121 °C for 20 min and thereafter inoculated with 1 mL of a mid-exponential phase pre-culture containing 4×10^6^ cells/mL. The cultures were incubated in a rotary shaker (ZHWY211C, Zhicheng, Shanghai, China) at 180 rpm and *T* = 28 ± 1 °C.

Determination of extracellular and intracellular lipids was performed as described in [2]. Intracellular lipids were fractionated as described in [3]. Fatty acid moieties of both extracellular and intracellular lipids and their fractions were converted into fatty acid methyl-esters (FAMEs) and analysed by using a Gas Chromatography (GC; Agilent 7890 A device, Agilent Technologies, Shanghai, China) as described in [4].

The predictions have been obtained using the mathematical model which is presented in [1].

**Fig. 1:**
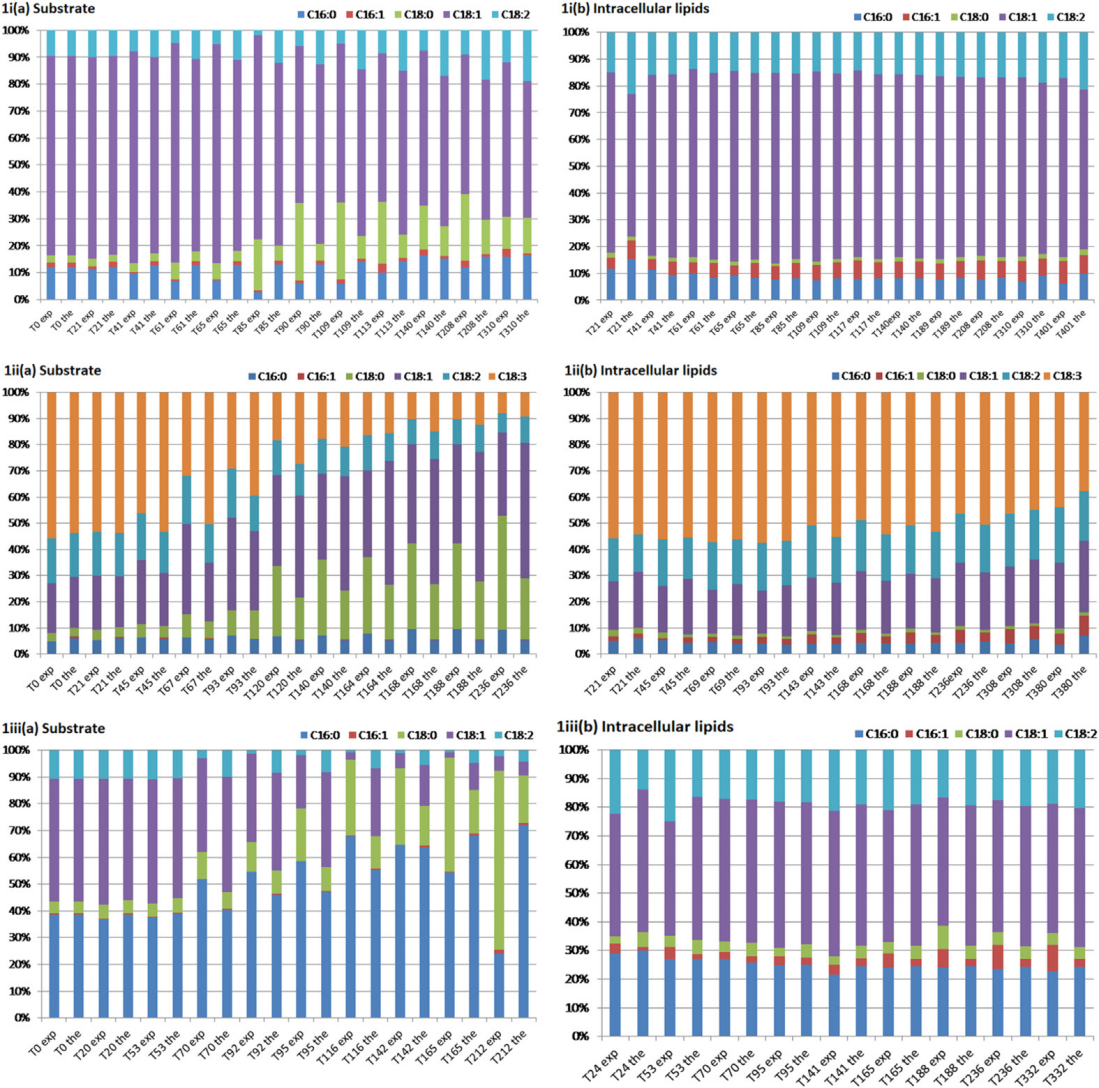

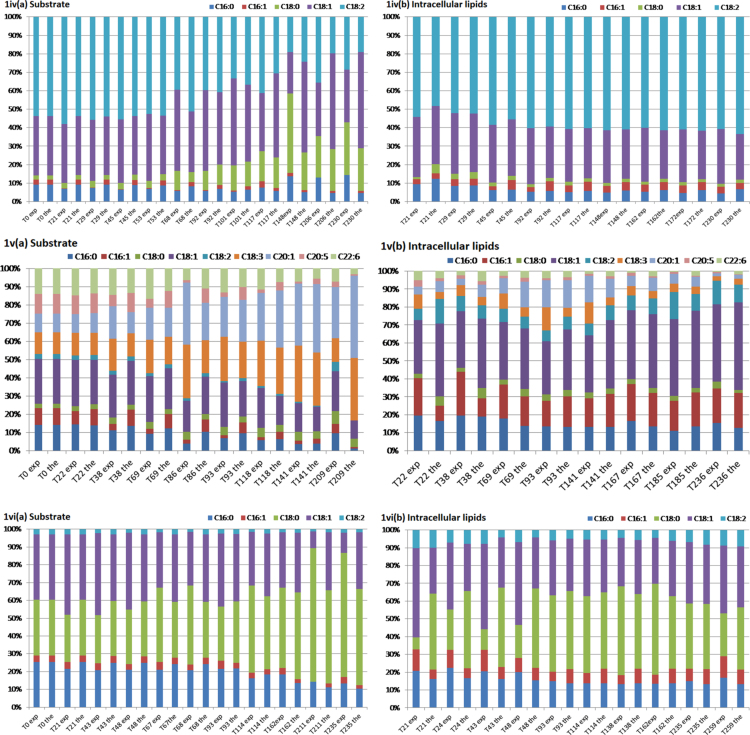
Experimental data and theoretical predictions of the fatty acid composition (%) of extracellular (a) and intracellular (b) lipids of *Yarrowia lipolytica* cultivated on: (i) Olive oil, (ii) linseed oil, (iii) palm oil, (iv) sunflower oil, (v) cod liver oil, and (vi) beef tallow. Culture conditions: As in Table 1.

**Fig. 2:**
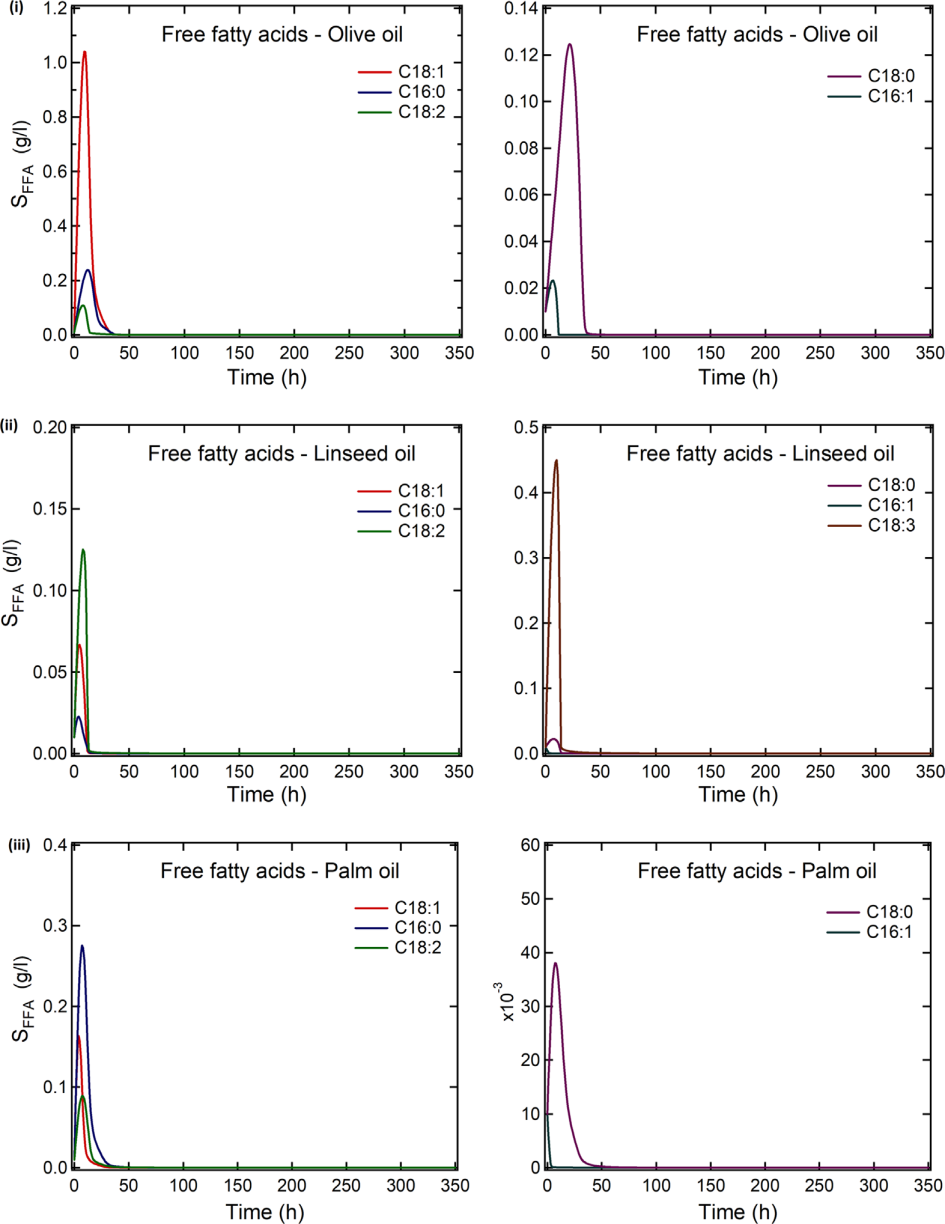

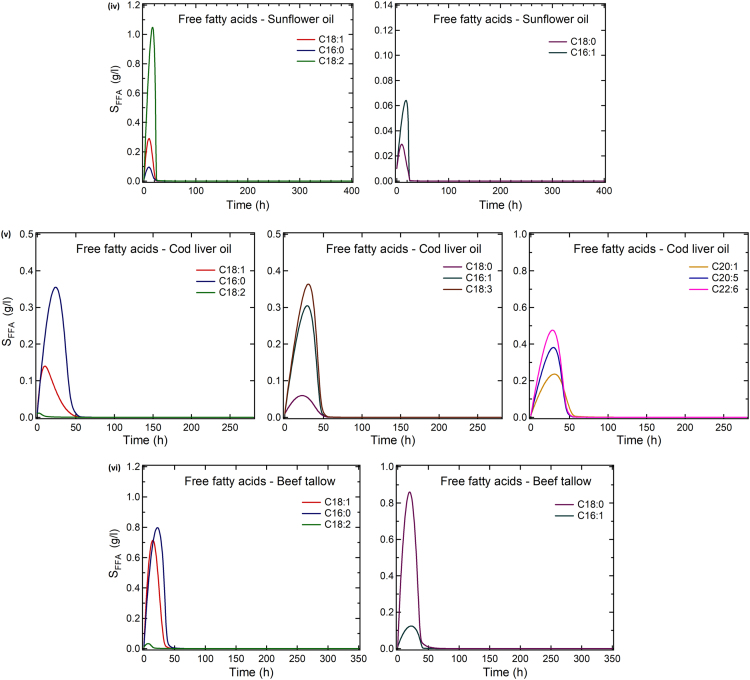
Theoretical fatty acid profiles of the free fatty acid fraction released in the growth medium (g/l) vs. time when *Yarrowia lipolytica* was cultivated on: (i) Olive oil, (ii) linseed oil, (iii) palm oil, (iv) sunflower oil, (v) cod liver oil, and (vi) beef tallow. Culture conditions: As in Table 1.

Experiments were performed in duplicate. Data represent means of two replicates. (Fig. 1, Fig. 2)
