# Supplementary material for: Data on cellular lipids of Yarrowia lipolytica grown on fatty substrates
Source: Data Brief. 2018 Oct 28;21:1037–44. doi: 10.1016/j.dib.2018.10.116 (PMC6226593; doi:10.1016/j.dib.2018.10.116)
Supplement: Supplementary file 1 — Supplementary material [file mmc1.docx]

Dear Editor,

I declare no conflict of interest.

G Aggelis
